# Neural network-based method for diagnosis and severity assessment of Graves’ orbitopathy using orbital computed tomography

**DOI:** 10.1038/s41598-022-16217-z

**Published:** 2022-07-15

**Authors:** Jaesung Lee, Wangduk Seo, Jaegyun Park, Won-Seon Lim, Ja Young Oh, Nam Ju Moon, Jeong Kyu Lee

**Affiliations:** 1 School of Computer Science and Engineering, Chung-Ang University, Seoul, Korea; 2 Department of Ophthalmology, Chung-Ang University College of Medicine, Chung-Ang University Hospital, 102 Heukseok-ro, Dongjak-gu, Seoul, 06973 Korea

**Keywords:** Biomarkers, Diseases, Medical research

## Abstract

Computed tomography (CT) has been widely used to diagnose Graves’ orbitopathy, and the utility is gradually increasing. To develop a neural network (NN)-based method for diagnosis and severity assessment of Graves’ orbitopathy (GO) using orbital CT, a specific type of NN optimized for diagnosing GO was developed and trained using 288 orbital CT scans obtained from patients with mild and moderate-to-severe GO and normal controls. The developed NN was compared with three conventional NNs [GoogleNet Inception v1 (GoogLeNet), 50-layer Deep Residual Learning (ResNet-50), and 16-layer Very Deep Convolutional Network from Visual Geometry group (VGG-16)]. The diagnostic performance was also compared with that of three oculoplastic specialists. The developed NN had an area under receiver operating curve (AUC) of 0.979 for diagnosing patients with moderate-to-severe GO. Receiver operating curve (ROC) analysis yielded AUCs of 0.827 for GoogLeNet, 0.611 for ResNet-50, 0.540 for VGG-16, and 0.975 for the oculoplastic specialists for diagnosing moderate-to-severe GO. For the diagnosis of mild GO, the developed NN yielded an AUC of 0.895, which is better than the performances of the other NNs and oculoplastic specialists. This study may contribute to NN-based interpretation of orbital CTs for diagnosing various orbital diseases

## Introduction

Graves’ orbitopathy (GO) is an autoimmune disorder that is mainly associated with Graves’ disease (GD). GO is primarily diagnosed based on its characteristic ophthalmic manifestations such as eyelid retraction, exophthalmos, and extraocular muscle (EOM) involvement, along with thyroid dysfunction^1^. However, inconclusive ophthalmic features in patients with euthyroidism or thyroiditis sometimes lead to an uncertain diagnosis^2^. No laboratory test or clinical findings pathognomonic for GO are available currently. Various diagnostic criteria for GO have been proposed^3^, but they remain controversial.

Computed tomography (CT) has been widely used to diagnose GO and determine appropriate treatment modalities. In the past, it was mainly used to provide supportive information for EOM enlargement when the clinical diagnosis was difficult. However, the application of CT to assess the degree of proptosis, compressive optic neuropathy, or even the activity of GO has been gradually increasing^4–7^. Despite the method’s increased usability, diagnosis of GO based on orbital CT findings had some shortcomings. It is difficult to identify changes in orbital fat using CT, and minimal changes in EOMs from normal variants can hardly be distinguished using this method. As there are no widely accepted studies on the normal variants of orbital tissues, it difficult to clearly distinguish orbital changes based on CT findings alone. Although several software programs are being developed for CT analysis^8,9^, but a more intuitive and easier method is needed to analyze CT findings and provide objective results for predicting the diagnosis and prognosis of patients with GO.

A neural network is an algorithm that aims to recognize underlying relationships in datasets through a process that imitates how the human brain works. Recently, several neural network (NN)-based methods have been developed for image analysis and were applied to diagnose various ocular disorders such as glaucoma, diabetic retinopathy, and age-related macular degeneration^10–13^. As the NNs were developed for general image analysis, they may be insufficient for diseases that require multiple specialized images to detect structural changes, such as orbital disorders. Diagnosis of diseases by applying NN-based methods to CT images is already being attempted for a few organs such as the lungs and brain^14,15^. However, NN-based techniques using orbital CT have rarely been investigated in diagnosing GO, and the usefulness remains to be unveiled. Therefore, in this study, we developed and evaluated a new NN that can be applied to GO diagnosis and severity assessment by analyzing orbital CT images.

## Results

The average age of the patients with GO was higher than that of the normal controls, and the average age of patients with moderate-to-severe GO was higher than that of the mild GO patients (p < 0.001). The gender ratios also differed among the groups, with a higher proportion of women in the control group. The margin reflex distance (MRD) 1 and exophthalmos were significantly different between the groups (*p* < 0.001). The MRD1 was significantly higher in moderate-to-severe GO patients than other groups, and significantly higher in mild GO patients than controls. The exophthalmos was significantly greater in moderate-to-severe GO patients than mild GO patients and controls. There were no significant differences in exophthalmos between mild GO patients and controls. Table 1 presents the demographic characteristics of the groups.

**Table 1:** Clinical and demographic characteristics of participants.

Characteristics	Mild GO	Moderate-to-severe GO	Controls	*p *value
Number of patients (N)	99	94	95	
Age (years)	38.4 ± 10.4	47.6 ± 15.0	29.3 ± 8.1	< 0.001*
Sex (male/female)	13/86	45/49	37/58	< 0.001**
Exophthalmos	16.7 ± 2.02	18.7 ± 3.27	17.2 ± 1.66	< 0.001*
MRD1	3.62 ± 1.18	4.22 ± 1.61	3.01 ± 1.09	< 0.001*

*MRD* margin reflex distance, *GO* Graves’ orbitopathy.

*One-way ANOVA test.

**Pearson’s chi-square test.

Table 2 presents the experimental results of the proposed and conventional NNs, reporting the values averaged over 30 repetitive experiments. The experimental results indicate that the proposed method achieved an AUC of 0.905 for moderate-to-severe GO patients vs. mild GO patients vs. normal controls. For the moderate-to-severe GO patients vs. normal controls, the proposed NN achieved an AUC of 0.979, while yielded a relatively low AUC of 0.895 for the mild GO patients vs. normal controls. It is interesting to note that the combination of all axial, coronal, and sagittal plane images produced higher diagnostic performance in terms of AUC than the use of CT images of only one or two planes. In addition, when using CT images of only one or two planes, the axial plane images tend to produce higher AUC compared with other planes for the mild GO patients vs. normal controls. On the other hand, using the sagittal plane images tends to produce higher AUC compared with other planes for the moderate-to-severe GO patients vs. mild GO patients and moderate-to-severe GO patients vs. mild GO patients vs. normal controls. We also validated the accuracy of the proposed NN. In the 30 repeated experiments, the proposed NN achieved accuracies of 0.930 for the moderate-to-severe GO patients vs. normal controls, 0.868 for the moderate-to-severe GO patients vs. mild GO patients, 0.826 for the mild GO patients vs. normal controls, and 0.842 for the moderate-to-severe GO patients vs. mild GO patients vs. normal controls.

**Table 2:** AUCs of diagnostic ability for Graves’ orbitopathy using NNs.

Model	CT plane	Moderate-to-severe GO vs. controls	Mild GO vs. controls	Moderate-to-severe GO vs. mild GO	Moderate-to-severe GO vs. mild GO vs. controls
Proposed model	Axial	0.920 ± 0.080**^▼^	0.849 ± 0.059*^▼^	0.843 ± 0.052**^▼^	0.781 ± 0.054**^▼^
	Coronal	0.956 ± 0.035*^▼^	0.760 ± 0.069**^▼^	0.855 ± 0.048**^▼^	0.797 ± 0.045**^▼^
	Sagittal	0.963 ± 0.029	0.821 ± 0.060**^▼^	0.932 ± 0.032	0.833 ± 0.059**^▼^
	Axial + coronal	0.973 ± 0.021	0.888 ± 0.049	0.892 ± 0.043**^▼^	0.865 ± 0.043**^▼^
	Axial + sagittal	0.971 ± 0.028	0.875 ± 0.058	**0.941 ± 0.035**	0.889 ± 0.044
	Coronal + sagittal	0.970 ± 0.029	0.821 ± 0.064**^▼^	0.935 ± 0.037	0.879 ± 0.043*^▼^
	Axial + coronal + sagittal	**0.979 ± 0.020**	**0.895 ± 0.052**	0.933 ± 0.041	**0.905 ± 0.029**
GoogLeNet	Axial	0.827 ± 0.135**^▼^	0.706 ± 0.091**^▼^	0.754 ± 0.133**^▼^	0.666 ± 0.065**^▼^
	Coronal	0.774 ± 0.161**^▼^	0.636 ± 0.063**^▼^	0.714 ± 0.119**^▼^	0.581 ± 0.027**^▼^
	Sagittal	0.710 ± 0.189**^▼^	0.800 ± 0.120**^▼^	0.632 ± 0.189**^▼^	0.673 ± 0.038**^▼^
ResNet-50	Axial	0.526 ± 0.070**^▼^	0.528 ± 0.084**^▼^	0.536 ± 0.111**^▼^	0.534 ± 0.047**^▼^
	Coronal	0.512 ± 0.091**^▼^	0.499 ± 0.005**^▼^	0.487 ± 0.058**^▼^	0.509 ± 0.025**^▼^
	Sagittal	0.611 ± 0.147**^▼^	0.526 ± 0.120**^▼^	0.491 ± 0.063**^▼^	0.580 ± 0.072**^▼^
VGG-16	Axial	0.495 ± 0.043**^▼^	0.512 ± 0.042**^▼^	0.499 ± 0.006**^▼^	0.508 ± 0.005**^▼^
	Coronal	0.504 ± 0.019**^▼^	0.498 ± 0.013**^▼^	0.499 ± 0.007**^▼^	0.508 ± 0.005**^▼^
	Sagittal	0.540 ± 0.096**^▼^	0.509 ± 0.058**^▼^	0.531 ± 0.083**^▼^	0.512 ± 0.009**^▼^

*AUC* area under the curve, *CT* computed tomography, *GO* Graves’ orbitopathy, *NN* neural network.

▼ indicates that the corresponding method is significantly worse than proposed model based on paired *t*-test. *▼: *p* < 0.01, **▼: *p* < 0.001.

Significant values are in bold.

The performance of GoogLeNet, which has the best diagnostic performance among the three conventional NNs, was much lower than that of the proposed model (*p* < 0.001). The AUC for discriminating between moderate-to-severe GO patients vs. mild GO patients vs. normal controls was 0.673. In the diagnosis of moderate-to-severe GO patients vs. normal controls, ROC curve analysis revealed 0.827 for GoogLeNet, 0.611 for ResNet-50, and 0.540 for VGG-16, respectively. Since each of the conventional NNs has only one input node, only single-plane CT images were selected for each analysis.

Furthermore, we specified an ablation study of NN structure by changing the primary components from a baseline NN structure until the proposed structure is achieved. Table 3 presents the results of the ablation study, reporting the AUC values averaged over ten repetitive experiments for the moderate-to-severe GO patients vs. mild GO patients vs. normal controls. Specifically, Stage1 NN is the baseline structure and consists of three standard convolutional layers and one fully connected layer, reporting baseline performance. Stage2 NN, including depthwise convolutions, exhibited considerable AUC improvement compared to Stage1 NN. Compared with the Stage2 NN, Stage3 NN improved AUC by including half depthwise convolution for orbit comparison. Finally, the proposed NN further improved AUC by separating the parameters of the half depthwise convolution into parts for the left and right orbits.

**Table 3:** Ablation study of proposed NN for Graves’ orbitopathy in terms of AUC.

Model	Description	Moderate-to-severe GO vs. mild GO vs. controls
Stage1 NN	All the 3 layers of NN were composed of standard convolutions	0.774 ± 0.128
Stage2 NN	The second and third layer’s convolutions of NN were replaced with depthwise convolutions	0.899 ± 0.020
Stage3 NN	Depthwise convolutions were replaced with half depthwise convolutions for the left and right orbits	0.902 ± 0.027
Proposed NN	Unlike the Stage3 NN, half depthwise convolutions for the left and right orbits were separately trained	**0.905 ± 0.029**

*AUC* area under the curve, *GO* Graves’ orbitopathy, *NN* neural network.

Significant values are in bold.

**Figure 1:**
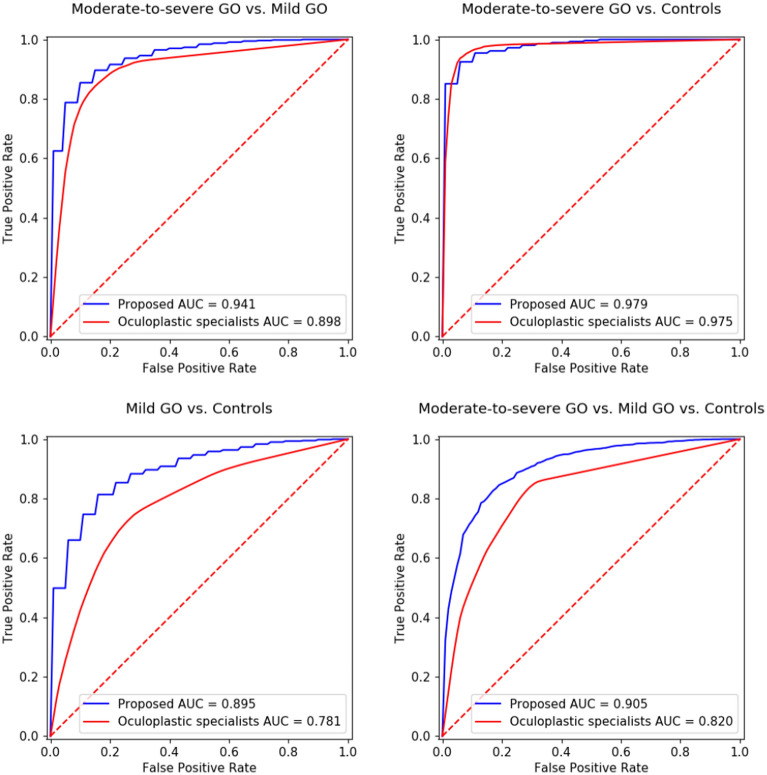
ROC curves of proposed NN and oculoplastic specialists. *GO* Graves’ orbitopathy, *AUC* area under the curve.

Figure 1 shows ROC curves representing the diagnostic performance of the oculoplastic specialists. The AUCs were determined to be 0.898 for the moderate-to-severe GO patients vs. mild GO patients, 0.975 for moderate-to-severe GO patients vs. normal controls, 0.781 for mild GO patients vs. normal controls, and 0.820 for moderate-to-severe GO patients vs. mild GO patients vs. normal controls. The experimental results indicate that the performance of the proposed NN is comparable to that of the oculoplastic specialists for the case of moderate-to-severe GO patients vs. normal controls.

**Figure 2:**
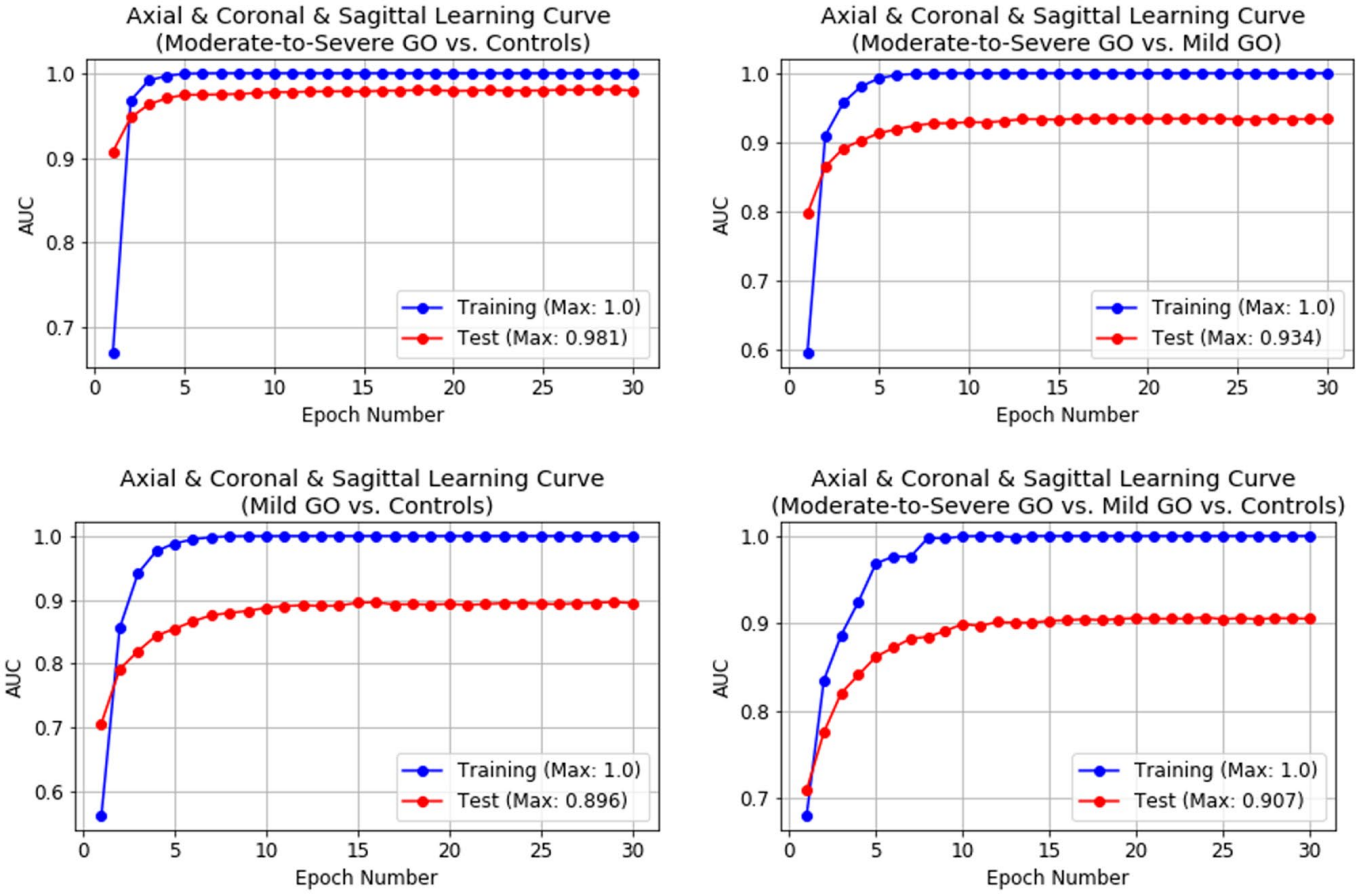
Learning curves of proposed neural network at each epoch averaged over 30 experiments. The blue and red lines correspond to the training and test datasets, respectively. Both lines in each case indicate the best performance at the 10th epoch without oscillation, and the area under the receiver operating characteristic curves (AUC) of all the learning curves increase monotonically. *GO* Graves’ orbitopathy.

In NN studies, it is important to review the learning curves of the models during training to diagnose learning issues such as overfitting. Figure 2 depicts the learning curves of the proposed NN at each epoch averaged over 30 experiments. To obtain reliable results, we used the repetitive holdout cross-validation with random split whenever conducting each experiment because the learning curves can vary according to test sets. The figure indicates stable learning that the learning curves increase monotonically and then reach the best performance at the 10th epoch without oscillation.

## Discussion

In a previous study, it has been reported that NN can be applied to the classification of GO patients and prognosis prediction^16^. Our results demonstrated the possibility of applying machine learning techniques to orbital CT images to discriminate patients with GO from normal controls effectively. The diagnosis of GO is usually unambiguous in patients with a history of GD and typical clinical features. However, if there are atypical features, CT or magnetic resonance imaging (MRI) is required to rule out other important diagnose. MRI can accurately reflect changes in soft tissue, but can be more expensive. The criteria for diagnosing GO patients with CT alone has not yet been established. However, there are many reports that abnormal findings such as extraocular muscle hypertrophy have been reported on CT images of GO patients^7,17^. Therefore, if GO patients can be accurately diagnosed using CT scan, the risk of missing a diagnosis or unnecessary treatment can be avoided. We developed a novel convolutional NN that is specialized for GO diagnosis, and the AUC for diagnosing moderate-to-severe GO patients in comparison with normal controls was found to be the highest at 0.979, while it was 0.941 for discriminating patients with moderate-to-severe and mild GO. In addition, the proposed NN demonstrated much higher performance than conventional NNs such as GoogLeNet, ResNet-50, and VGG-16. The performance of the proposed NN was comparable to or even higher than that of oculoplastic specialists, introducing the possibility of clinical use in ophthalmic practice.

Our proposed NN contained three convolutional layers with three input channels followed by one fully connected layer and a final sigmoid classification layer for binary classification and softmax layer for multi-classification. Considering that more than 100 CT images with different planes must be analyzed for each patient, the configuration of multiple input channels ensures that all relevant features are available without loss^18^. The proposed NN has main differences compared with the conventional NNs. First, the proposed NN could improve the diagnostic performance by including information processing pipelines for CT images of each plane and combining them, a technique not implemented in other NNs. Second, the proposed NN involves a novel process of binocular comparison that may be beneficial for diagnosing other orbital diseases. Meanwhile, the features in the proposed NN are extracted with two-dimensional (2D) approaches rather than three-dimensional (3D) techniques. Since CT images are 3D, 2D techniques may lack 3D spatial and volume information significant for classification. Nevertheless, most convolutional NNs so far have been designed for natural 2D images^19^. A comparison of 3D and 2D convolutional NNs will be necessary in the future.

The NNs were less effective in distinguishing patients with mild GO from normal controls than patients with moderate-to-severe GO form normal controls. This result may be due to the limitation of relying solely on orbital CT findings to diagnose mild GO patients. Although there have been no reports of the diagnostic accuracy of orbital CT alone for mild GO, this approach may not be accurate in diagnosing mild GO with eyelid retraction of less than 2 mm or exophthalmos less than 3 mm. As such patients are usually diagnosed based on clinical findings rather than orbital CT, NN using orbital CT images may be less effective. Nevertheless, the proposed NN showed higher diagnostic performance than oculoplastic specialists in discriminating patients with mild GO from normal controls. Substantial inconsistency in image assessment among observers has been reported in previous studies, and the classification of CT findings for orbital diseases may also be prone to high intra- and inter-observer fluctuations^20,21^. Therefore, it can be assumed that NNs are able to detect subtle and early changes that are difficult for humans to judge visually or consistently. Mild GO patients are often misdiagnosed as normal when their symptoms are not typical, and these patients can benefit most from NN in the diagnostic process. NNs can be helpful when there is a need to determine whether a patient has mild GO, such as when clinical features are inconclusive or when establishing a Graves’ disease treatment policy.

Overfitting, in which a model is trained too well and represents a poor model for unseen data, is a common issue when training a model with a small dataset. Most medical data are limited in number due to prevalence and acquisition costs, and overfitting was also an issue in our study. To overcome this problem, we reduced the number of features by preprocessing and validated the learning procedure using cross-validation and a learning curve. When these methods were applied, the validation metric improved until a certain number of epochs was reached, then remained constant without decreasing, suggesting that no overfitting occurred.

Although we developed an NN, it is difficult to elucidate how the NN classifies orbital CT images as normal or GO. We attempted to evaluate the logic structure through which the NN works by classifying the feature map patterns, but owing to the nature of NNs as black boxes, no further tracking was possible. Previous studies have reported that changes in the size of the extraocular muscle or lacrimal gland in orbital CT are important for predicting activity or severity in GO patients^17,22^. It is hard to know whether the NN classified orbital images according to changes in extraocular muscle or lacrimal gland in our study. It may be necessary to infer the diagnostic process of NN through segmentation of intra-orbital tissues and supervised training. Interpretability is important, and if it is impossible for a clinician to verify the logical mechanism or approach of NN, it is difficult to accept computer-aided diagnosis using NNs in clinical practice^19^. Therefore, further study is needed to visualize the convolutional layers and filters to form an idea of how machines classify images.

Our study has several limitations. First, there could have been selection bias due to the nature of the datasets, which were selected from a single tertiary hospital. As there is no standardized dataset of orbital CTs for GO, it was necessary to rely on the available hospital data. Further, the normal controls included patients who were evaluated for exophthalmos; therefore, the controls may not have accurately represented the normal population owing to the inclusion of patients with high myopia. However, there was no significant difference in the exophthalmos between mild GO patients and controls. Considering the fact that it is difficult to obtain an orbital CT scan of a normal subject without any ocular symptoms, the controls can be designated as a normal control group. Furthermore, this study was based on only one imaging modality. The current algorithm does not incorporate clinical information. The severity of GO is mainly determined by clinical findings, and there are no criteria for orbital CT diagnosis. Therefore, if other information such as age, sex, and clinical pictures are also used for judgment, further improvements in diagnostic performance can be expected.

In conclusion, we demonstrated the applicability of NNs to diagnosis of GO and its severity assessment using clinically routine orbital CTs. The proposed NN can reliably distinguish patients with moderate-to-severe GO from normal controls, although it is less effective in discriminating between patients with mild GO patients and normal controls. The performance of our technique is comparable to that of oculoplastic specialists. This research may contribute to NN-based interpretation of orbital CTs for diagnosing various orbital diseases. The code is publicly available at https://github.com/laymond1/Graves-Orbitopathy-Diagnosis-using-Neural-Network. Further research is necessary to automate orbital CT analysis to develop a diagnosis system that is fully independent of human effort.

## Materials and methods

The Institutional Review Board (IRB) of Chung-Ang University Hospital approved this study. This was a retrospective study, and the requirement for informed consent was waived by the IRB of Chung-Ang university Hospital (IRB No. 1912-004-358). This study was conducted in accordance with the ethical standards outlined in the Declaration of Helsinki.

### Study participants

The orbital CTs used in this study were obtained from 200 patients with GO and 100 normal controls between December 2010 and December 2018. The GO patients were diagnosed based on Bartley’s criteria^23^, and the severity of GO (mild or moderate-to-severe) was evaluated according to the severity scale of the European Group on Graves Orbitopathy (EUGOGO) consensus^24^. The patients with mild GO have one or more of the following: minor lid retraction (< 2 mm), mild soft-tissue involvement, exophthalmos < 3 mm above normal, no or intermittent diplopia and corneal exposure responsive to lubricants. The patients with moderate-to-severe GO were defined as those with more severe clinical features without visual impairment than patients with mild GO. Normal controls were participants who visited the clinic for exophthalmos evaluation without any disease history and were confirmed to have no disease other than myopia. Exclusion criteria were an age of 18 years or less, previous orbital surgery, axial length greater than 27 mm in either eye, and other orbital pathology that may affect CT findings including myasthenia grave and progressive external ophthalmoplegia. The data of the patients including age, sex, MRD1, and exophthalmometry were recorded for analysis. CT images were obtained with one of the three following scanners: Brilliance CT 64 (Philips Medical Systems, Cleveland, OH, USA), Optima CT660 Freedom Edition (General Electric Medical Systems, Milwaukee, WI, USA), or IQon Elite Spectral CT (Philips Medical Systems, Cleveland, OH, USA). Continuous scanning with a slice thickness of 1.0 mm and slice increment of 1.0 mm was performed. All images were deidentified prior to transfer to the study investigators.

The CT images were jointly evaluated by an ophthalmologist and a radiologist, and images that were incomplete or inconsistent with the clinical findings were excluded. In total, 288 CT image sets were obtained, including 99 cases of mild GO, 94 of moderate-to-severe GO, and 95 of normal controls.

### Data preparation

**Figure 3:**
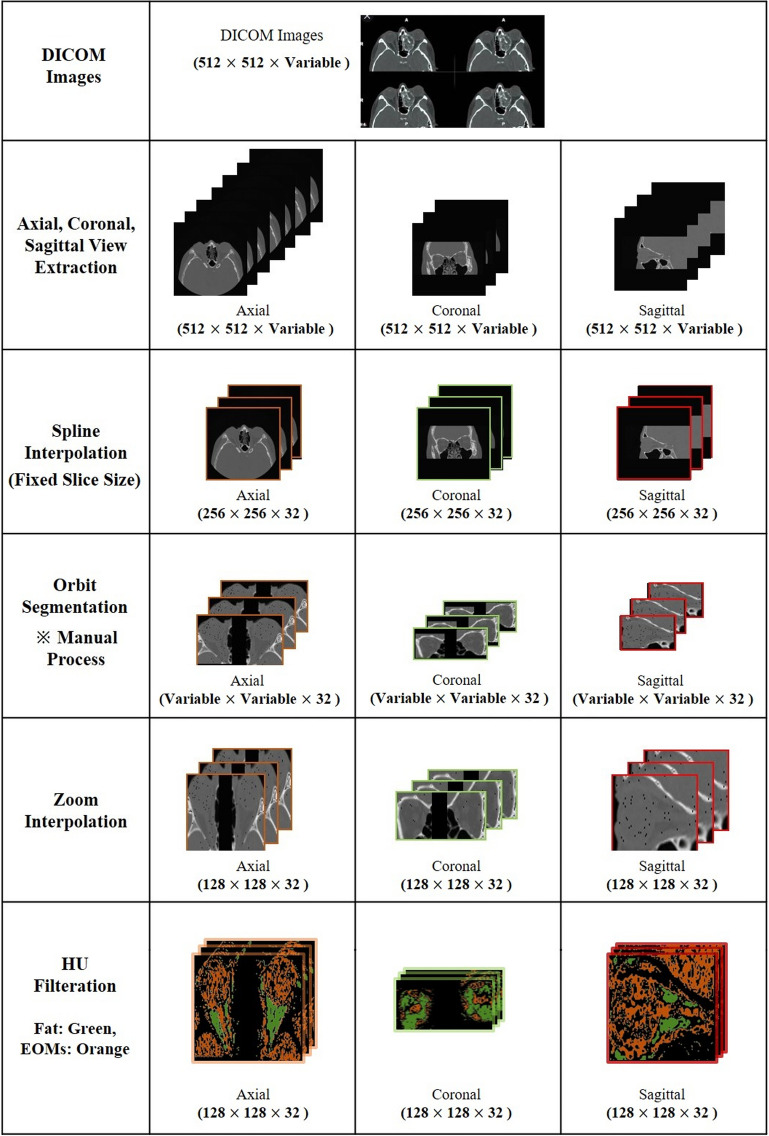
Data preparation process. The soft tissue thresholds were set at −100 to + 40 attenuation values in Hounsfield units (HU) to remove unnecessary pixels. Manual cropping was performed, and the extracted region of interests (ROI) were unified in size by interpolation.

The obtained CT images in the axial, coronal, and sagittal planes were uploaded to a RadiAnt DICOM viewer (Medixant Co., Poznan, Poland). To overcome the variations caused by differences in CT equipment, spline interpolation was used to fix the number of images in each plane to 32. Then, we manually cropped the region of interest (ROI) and removed the remaining black margin. To meet the fixed-size input requirement for NNs, the CT images were then zoom-interpolated, enlarging the region of interest, to 128 × 128 for the axial (128, 128, 32) and sagittal (128, 128, 32) planes, and 64 × 128 for the coronal plane (64, 128, 32). The CT images were scaled to Hounsfield unit (HU) values, and fat and EOMs were selected in the ranges of −110 to −10, and 0–40 HU, respectively, to remove unnecessary pixels^17^. Finally, all images were normalized by scaling between 0 and 1. Figure 3 shows a schematic overview of our preprocessing steps.

All 288 cases were separated and combined into four experimental groups: (1) moderate-to-severe GO vs. normal controls, (2) mild GO vs. normal controls, (3) moderate-to-severe GO vs. mild GO, and (4) moderate-to-severe GO vs. mild GO vs. normal controls. Next, each experimental group was represented as an isolated dataset. To mitigate the effects of selection bias due to sex and age, we used holdout cross-validation with random split regardless of the clinical or demographic characteristics of the participants; for each dataset, 80% were used as a training set to train the NNs and the remaining 20% were used as a test set to evaluate the trained NNs. The final performances of the proposed NN and existing NNs were measured by averaging the results of 30 repetitive experiments.

### Convolutional NN

**Figure 4:**
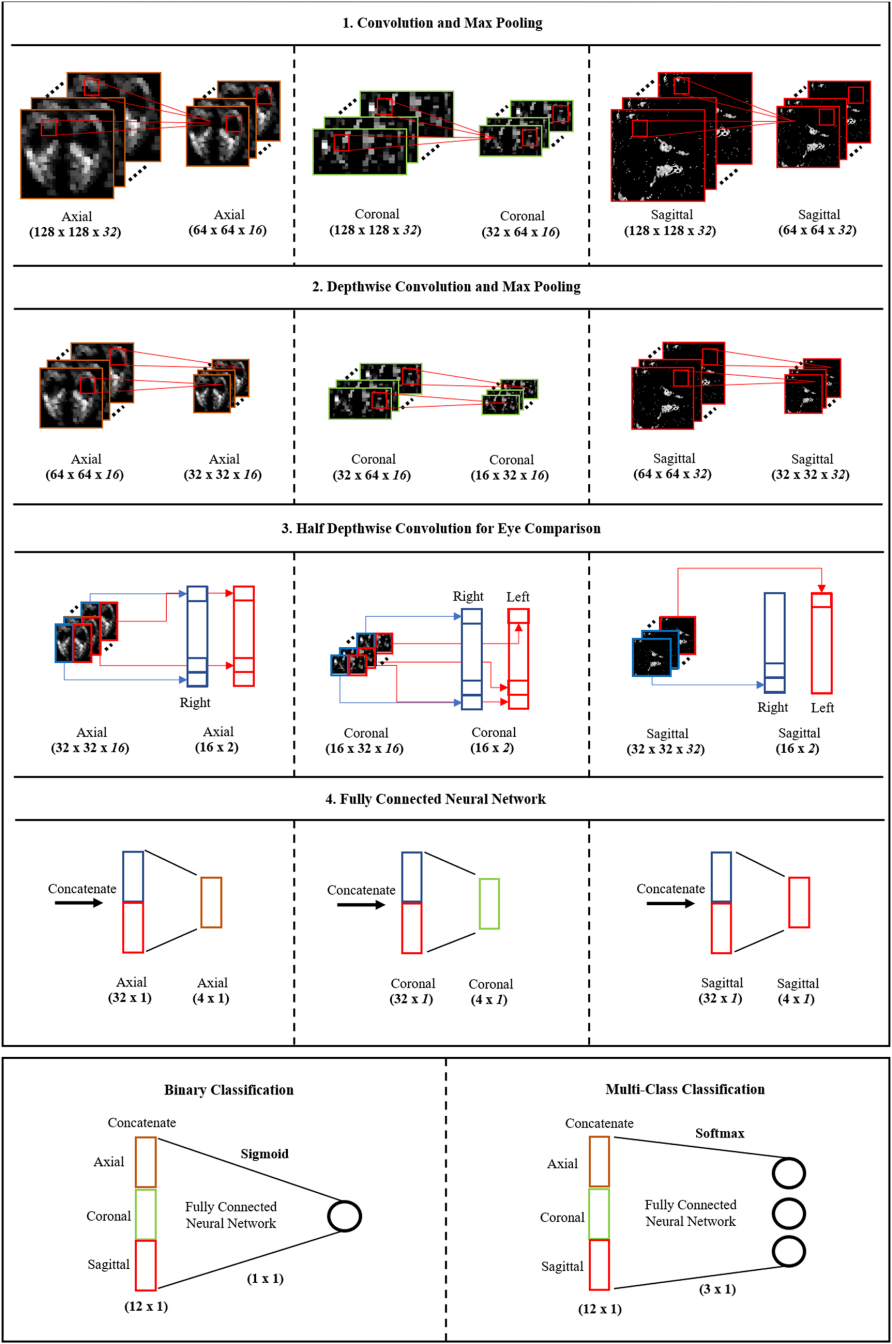
Overview of neural network modeling. The neural network consists of convolutional operators with a half depth-wise convolution layer for binocular comparison that reduces the number of parameters compared to that in a convolutional neural network.

In practice, it is preferable to consider CT images from axial, coronal, and sagittal planes because they deliver different information for diagnosing GO. However, conventional NN is designed to accept three-channel inputs such as RGB color images. Although a single image plane may be handled by increasing the number of input channels of existing NNs, the conventional NNs are unable to utilize these three image planes concurrently with their original input layer. To deal with this issue, we design a new NN that is able to accept three image planes concurrently. Figure 4 shows an overview of the proposed NN. Each cell describes the behavior of the operator and the shapes of input and output nodes. The proposed NN has three input layers consisting of 32-bit single-precision floating point elements that take axial (128 × 128 × 32), sagittal (128 × 128 × 32), and coronal images (64 × 128 × 32), which are handled independently before the final fully connected layer. In the proposed NN, first, features are extracted from convolutional and depth-wise convolutional layers based on the input CT images of the maximum three different planes. A max pooling layer follows each convolution operation. After the first step, the sizes of the axial and coronal images are reduced to 32 × 32 × 16 and 16 × 32 × 16, respectively. Since only one orbit is included in each sagittal image, the reduced size (32 × 32 × 32) was larger than those of the axial and coronal CT images. Next, feature maps are separately extracted from the left and right orbits of each image to compare the orbits to detect asymmetry or unilateral GO. For this purpose, we use depthwise convolution, where the filter size was set to half of the image size. Each convolutional filter extracts a real value from the separated left or right orbit image. Specifically, 16 convolutional filters are used for each orbit to produce the input values for the subsequent 16 × 2 nodes. Third, each group of 16 × 2 nodes is flattened to 32 × 1 nodes, which are fully connected to the subsequent 4 × 1 nodes. Finally, the output values of 4 × 1 nodes are transferred to the output layer. The output layer includes one sigmoid node to calculate the significance value if the dataset consists of two classes, such as mild GO patients vs. normal controls. For multi-class classification, such as moderate-to-severe GO patients vs. mild GO patients vs. normal controls, the output layer includes three softmax nodes.

**Figure 5:**
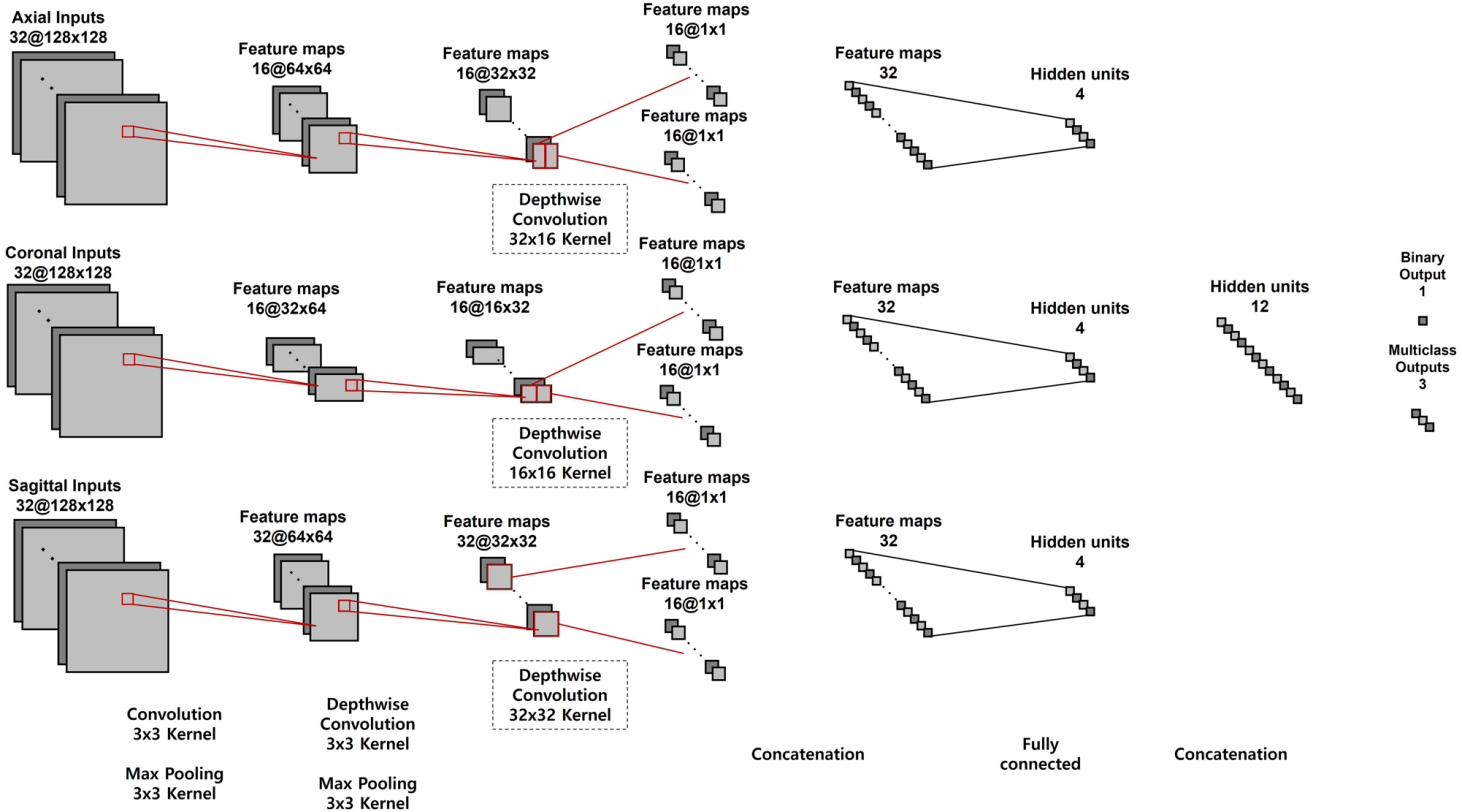
The schematic block diagram of the proposed NN. The size of feature maps and the types of operations for each layer are described sequentially according to the data flow.

Figure 5 shows a schematic block diagram of the proposed NN. As shown in Fig. 5, the proposed NN is divided into three parts of convolution layers, the fully connected layer, and the classifier. The parts up to the fully connected layer are calculated independently for each plane, and after concatenation, the extracted features are combined and used for prediction. In addition, Supplementary Fig. S1 visualizes the learned convolutional filters of the proposed NN. For simplicity, only the first 10 filters from each CT plane are shown, and the half depthwise convolutional filters are visualized by calculating the average for each of eight-by-eight grid areas.

### NN evaluation

The performance of the proposed NN was evaluated by comparing it with three conventional NNs: GoogLeNet Inception v1 (GoogLeNet), 50-layer Deep Residual Learning (ResNet-50), and 16-layer Very Deep Convolutional Network from Visual Geometry group (VGG-16)^25–27^. The NNs were implemented using the Tensorflow (2.1.0) and Keras (2.3.1) APIs, and overall experiments were executed on a GTX 1080Ti 11 GB GPU. For a fair comparison, we used the well-known Xavier initialization method supported as default in the APIs for all networks^28^. Specifically, because conventional NNs can train only one of image planes, we reported the performance values for the conventional NNs when CT images in a single plane were used as the input data. Each sample in the ImageNet dataset has 3 channels, which is RGB color scale, on the other hand, each sample of the medical data used in the experiment has 32 input channels. Thus, these convolutional networks were trained from scratch.

For comparison, three oculoplastic specialists were asked to perform four independent experiments in which they compared three experimental groups with full CT sets without any clinical information, as was done with the proposed and conventional NNs. The final grading was decided by the majority, and the diagnostic performance in terms of the area under the receiver operating characteristic (ROC) curve was compared with that of the proposed NN.

### Statistical analysis

All statistical analyses were performed using the open-source software R 3.4.0 (R Foundation for Statistical Computing, Vienna, Austria). The data were expressed as averages with standard deviations for the continuous variables and as sample numbers for the categorical variables. The differences in age, sex, and clinical features between the clinical groups were analyzed by one-way analysis of variance with Games-Howell post-hoc analyses and Pearson’s chi-square test, respectively. A value of *p* < 0.05 was considered statistically significant. Receiver operating characteristic (ROC) curve analysis was performed to evaluate the diagnostic performances of the various NNs and those of the oculoplastic specialists in terms of area under the ROC curve (AUC).

## Supplementary Information

Supplementary Figure S1.

## Data Availability

The datasets used and analysed during the current study are available from the corresponding author on reasonable request.

## References

[1] Naik VM, Naik MN, Goldberg RA, Smith TJ, Douglas RS (2010). Immunopathogenesis of thyroid eye disease: Emerging paradigms. Surv. Ophthalmol..

[2] Feldon SE (1990). Graves' ophthalmopathy. Is it really thyroid disease?. Arch. Intern. Med..

[3] Bartalena L (2016). The 2016 European thyroid Association/European group on Graves' orbitopathy guidelines for the management of Graves' orbitopathy. Eur. Thyroid. J..

[4] Nkenke E (2003). Relative en- and exophthalmometry in zygomatic fractures comparing optical non-contact, non-ionizing 3D imaging to the Hertel instrument and computed tomography. J. Craniomaxillofac. Surg..

[5] Feldon SE, Lee CP, Muramatsu SK, Weiner JM (1985). Quantitative computed tomography of Graves' ophthalmopathy: Extraocular muscle and orbital fat in development of optic neuropathy. Arch. Ophthalmol..

[6] Ramli N, Kala S, Samsudin A, Rahmat K, Abidin ZZ (2015). Proptosis-correlation and agreement between Hertel exophthalmometry and computed tomography. Orbit.

[7] Byun JS, Moon NJ, Lee JK (2017). Quantitative analysis of orbital soft tissues on computed tomography to assess the activity of thyroid-associated orbitopathy. Graefes Arch. Clin. Exp. Ophthalmol..

[8] Regensburg NI (2008). A new and validated CT-based method for the calculation of orbital soft tissue volumes. Invest. Ophthalmol. Vis. Sci..

[9] Souza AD, Ruiz EE, Cruz AA (2007). Extraocular muscle quantification using mathematical morphology: A semi-automatic method for analyzing muscle enlargement in orbital diseases. Comput. Med. Imaging Graph..

[10] Litjens G (2017). A survey on deep learning in medical image analysis. Med. Image. Anal..

[11] Gulshan V (2016). Development and validation of a deep learning algorithm for detection of diabetic retinopathy in retinal fundus photographs. JAMA.

[12] Shibata N (2018). Development of a deep residual learning algorithm to screen for glaucoma from fundus photography. Sci. Rep..

[13] Burlina PM (2018). Use of deep learning for detailed severity characterization and estimation of 5-year risk among patients with age-related macular degeneration. JAMA Ophthalmol..

[14] Röhrich S, Schlegl T, Bardach C, Prosch H, Langs G (2020). Deep learning detection and quantification of pneumothorax in heterogeneous routine chest computed tomography. Eur. Radiol. Exp..

[15] Chilamkurthy S (2018). Deep learning algorithms for detection of critical findings in head CT scans: A retrospective study. Lancet.

[16] Salvi M, Dazzi D, Pellistri I, Neri F, Wall JRJO (2002). Classification and prediction of the progression of thyroid-associated ophthalmopathy by an artificial neural network. Ophthalmology.

[17] Regensburg NI, Wiersinga WM, Berendschot TT, Saeed P, Mourits MP (2011). Densities of orbital fat and extraocular muscles in graves orbitopathy patients and controls. Ophthalmic. Plast. Reconstr. Surg..

[18] Gao M (2018). Holistic classification of CT attenuation patterns for interstitial lung diseases via deep convolutional neural networks. Comput. Methods Biomech. Biomed. Eng. Imaging Vis..

[19] Domingues I (2019). Using deep learning techniques in medical imaging: A systematic review of applications on CT and PET. Artifi. Intell. Rev..

[20] Colquhoun P (2003). Interobserver and intraobserver bias exists in the interpretation of anal dysplasia. Dis. Colon. Rectum..

[21] Scott IU (2008). Agreement between clinician and reading center gradings of diabetic retinopathy severity level at baseline in a phase 2 study of intravitreal bevacizumab for diabetic macular edema. Retina.

[22] Hallin ES, Feldon SE (1988). Graves' ophthalmopathy: II. Correlation of clinical signs with measures derived from computed tomography. Br. J. Ophthalmol..

[23] Bartley GB, Gorman CA (1995). Diagnostic criteria for Graves' ophthalmopathy. Am. J. Ophthalmol..

[24] Bartalena L (2008). Consensus statement of the European Group on Graves' orbitopathy (EUGOGO) on management of GO. Eur. J. Endocrinol..

[25] Szegedy, C., Vanhoucke, V., Ioffe, S., Shlens, J. & Wojna, Z. Rethinking the inception architecture for computer vision. *Proc. IEEE Comput. Soc. Conf. Comput. Vis. Pattern Recognit.* **2016**, 2818–2826 (2016).

[26] Conneau, A., Schwenk, H., Barrault, L. & Lecun, Y. Very deep convolutional networks for text classification. *arXiv preprint *arXiv:1606.01781 (2016).

[27] Wu S, Zhong S, Liu Y (2018). Deep residual learning for image steganalysis. Multimed. Tools Appl..

[28] Glorot X, Bengio Y (2010). Understanding the difficulty of training deep feedforward neural networks. Int. Conf. Artif. Intell. Stat..

