# Supplementary material for: Neural network-based method for diagnosis and severity assessment of Graves’ orbitopathy using orbital computed tomography
Source: Sci Rep. 2022 Jul 15;12:12071. doi: 10.1038/s41598-022-16217-z (PMC9287334; doi:10.1038/s41598-022-16217-z)
Supplement: Supplementary file 1 — Supplementary Figure S1. [file 41598_2022_16217_MOESM1_ESM.docx]

**Neural Network-based Method for Diagnosis and Severity Assessment of Graves’ Orbitopathy using Orbital Computed Tomography**

Jaesung Lee, PhD^1^, Wangduk Seo^1^, Jaegyun Park^1^, Won-Seon Lim^1^, Ja Young Oh, MD^2^, Nam Ju Moon, MD, PhD^2^, Jeong Kyu Lee, MD, PhD^2*^


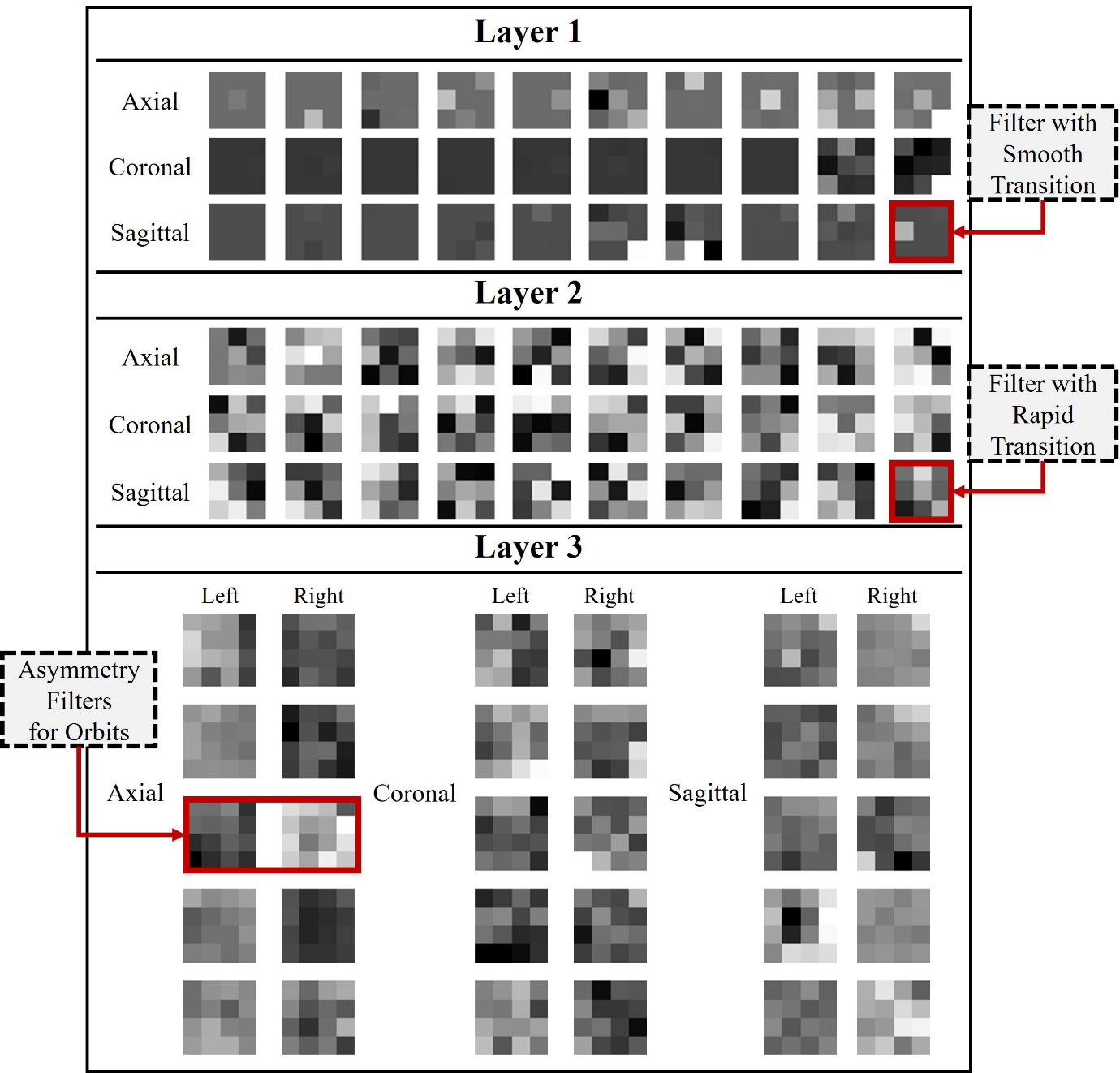


**Supplementary Figure S1.** The trained convolutional filters. In layer 1, we notice that the first convolution captures coarse-grained information such as edge detection. On the other hand, in layer 2, depthwise convolution tends to focus on more fine-grained information than that of layer 1. Moreover, in layer 3, half depthwise convolution focuses on asymmetric information for orbits at some input channels.
